# Macrophage Activation Syndrome Secondary to Underlying Sarcoidosis

**DOI:** 10.7759/cureus.4929

**Published:** 2019-06-18

**Authors:** Saro Kasparian, Kartik Anand, Ethan Burns, Betty Chung, Sai Ravi Kiran Pingali

**Affiliations:** 1 Internal Medicine, Houston Methodist Hospital, Houston, USA; 2 Hematology / Oncology, Houston Methodist Cancer Center, Houston, USA; 3 Pathology, Houston Methodist Hospital, Houston, USA

**Keywords:** macrophage activation syndrome, extrapulmonary sarcoidosis, sarcoid, treatment, hlh

## Abstract

Hemophagocytic lymphohistiocytosis (HLH) due to an underlying rheumatologic condition is known as macrophage activation syndrome (MAS), a rare and serious complication that often has a delayed diagnosis. MAS can complicate any rheumatologic disease, although it is most prevalent in systemic juvenile idiopathic arthritis. MAS occurring as a sequela of sarcoidosis is seldom reported. Herein, we present an uncommon case of MAS occurring secondary to suspected extrapulmonary sarcoidosis and the associated diagnostic challenges. A 53-year-old White female presented with a 20-month history of constitutional symptoms of an unclear etiology. Her extensive workup included equivocal bone marrow and liver biopsies, suggestive of occasional hemophagocytosis. On admission, she met criteria for HLH based on the HLH-94 diagnostic guidelines. A repeat liver biopsy was performed revealing non-necrotizing granulomas in the parenchyma. Given the concern for an extrapulmonary sarcoidosis, she was started on pulse-dose steroids with subsequent symptomatic resolution. Two years later, she remains in complete remission. As a systemic disease, sarcoidosis can manifest in any organ and present in a variety of ways. While HLH and MAS have numerous etiologies, sarcoidosis should be considered as a potential underlying diagnosis, and prompt treatment initiation with steroids may reduce morbidity and mortality.

## Introduction

Macrophage activation syndrome (MAS) is a potentially fatal syndrome of rheumatologic origin due to overactivation of cytotoxic CD8+ T-lymphocytes and macrophages. This process leads to an overproduction of proinflammatory cytokines, resulting in hemophagocytosis, tissue infiltration, and life threatening end-organ damage [1]. It is part of a spectrum of histiocytic disorders exhibiting hemophagocytic activity referred to as hemophagocytic lymphohistiocytosis (HLH). HLH is further delineated into primary (familial) or secondary (acquired) causes, with the etiology of the latter often due to infections, malignancies, or rheumatologic diseases [2]. A rapid onset and delayed clinical recognition may prolong the time to treatment initiation, contributing to a mortality rate of 20%-30% [3]. Previously considered a rheumatologic complication of pediatric patients, it is becoming apparent that MAS also occurs in adults with rheumatologic diseases, particularly systemic lupus erythematosus (SLE), adult onset Still’s disease, and rheumatoid arthritis [4]. Although seldom reported, MAS has also been seen with sarcoidosis. The following case adds to the limited medical literature on the association of MAS and sarcoidosis.

## Case presentation

**Figure 1:**
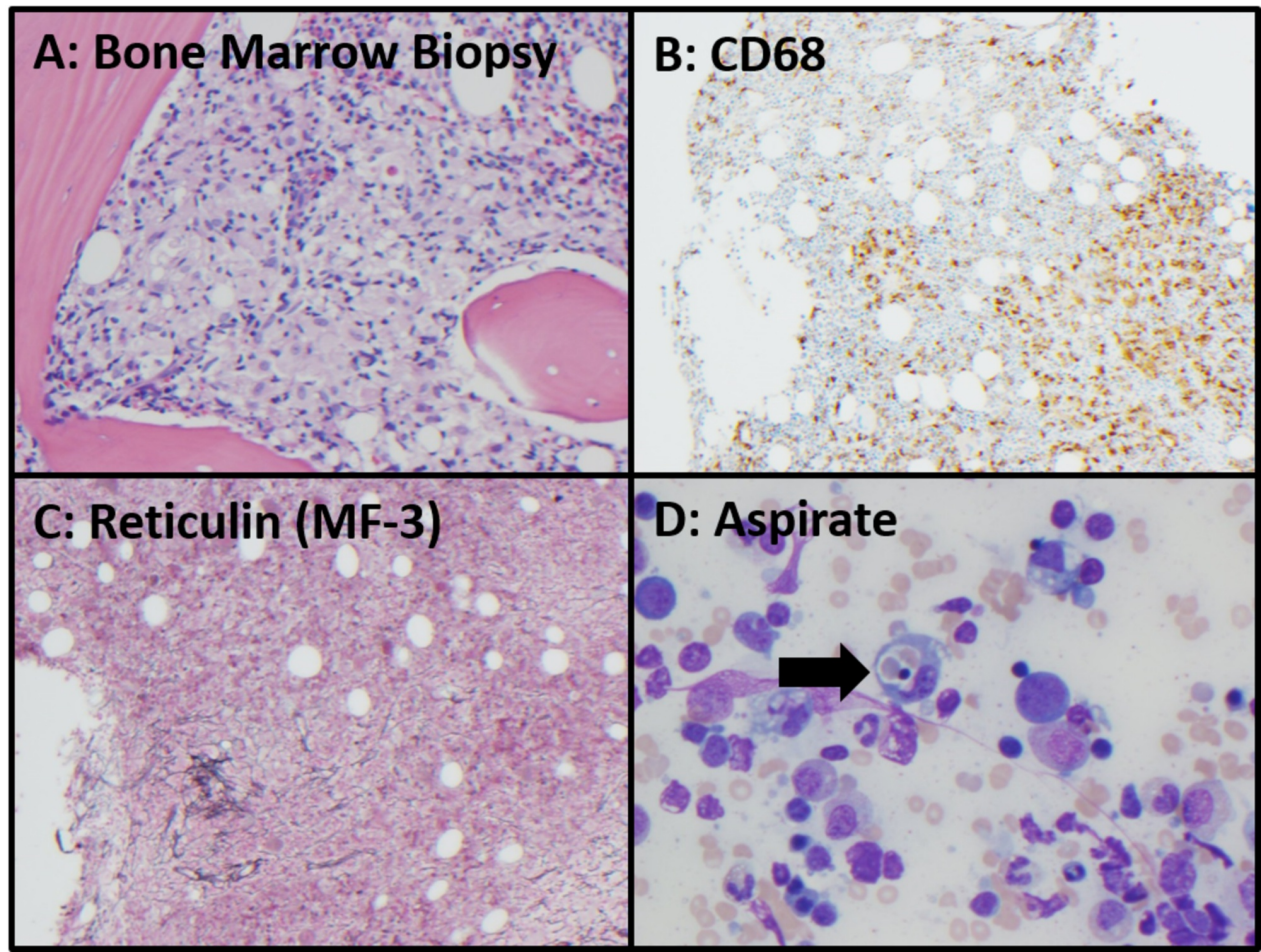
Bone Marrow Biopsy A: Ill-defined lymphohistocytic aggregates in core biopsy, 200x, hematoxylin and eosin. B: CD68 immunohistochemical stain highlights histiocytic cell infiltrates in marrow core biopsy, 100x. C: Reticulin special stain shows focal extensive reticulin fibrosis (grade MF-3), 100x. D: modified Giemsa-stained aspirate shows rare hemophagocytosis (arrow), 400x.

A 53-year-old White female with no medical history was referred to our institution after a 20-month history of progressive weakness, fatigue, night sweats, and fevers. Prior to admission, her physical examinations were remarkable for hepatosplenomegaly (HSM). Serial blood work was notable for prominent leukocytosis; however, studies for infectious causes including cytomegalovirus, human immunodeficiency virus, hepatitis, and bacteremia were negative. While she reported a history of Epstein-Barr Virus (EBV) infection in childhood, her EBV serum polymerase chain reaction (PCR) was negative. Antinuclear antibody testing was also negative. Imaging with ultrasonography and computed tomography (CT) reported HSM, without other significant findings. Over the course of 20 months, she developed pancytopenia. She underwent a bone marrow biopsy that revealed hemophagocytosis on aspirate, a hypercellular marrow, increased CD68+ histiocytes, and increased cytotoxic CD8+ T-cells. Flow cytometry and immunohistochemical (IHC) studies were suggestive of T-cell lymphocytosis but did not show findings of leukemia, non-Hodgkin's lymphoma, or plasma cell neoplasms. Cytogenetic studies revealed a normal karyotype. T-cell receptor (TCR) beta and gamma gene rearrangement studies revealed a monoclonal population of CD8+ T-lymphocytes. Given the patient’s HSM and clinical picture, she had a liver biopsy that revealed scattered portal lymphohistiocytosis and an infiltrative cytotoxic T-cell population. Additionally, a second bone marrow biopsy revealed lymphohistiocytic aggregates and rare hemophagocytosis (Figure 1).

**Figure 2:**
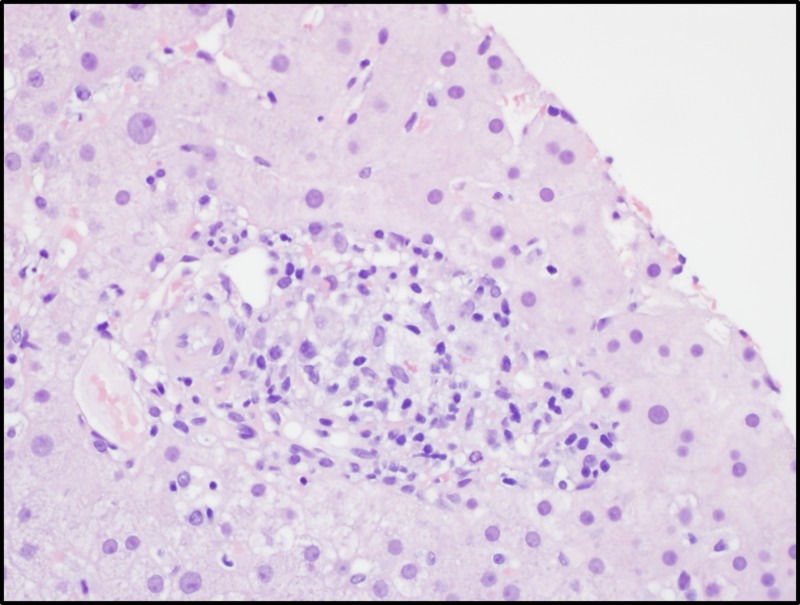
Liver Biopsy Portal and lobular non-necrotizing granulomata (consistent with granulomatous hepatitis) with patchy lobular lymphohistiocytic inflammation, patchy sinusoidal lymphocytes, and moderate mixed chronic periportal inflammation, 400x, hematoxylin and eosin stain.

Following this workup, she was referred to our institution. On presentation, her vitals were significant for persistent fevers and physical examination indicated HSM. Laboratory analysis was significant for bicytopenia with hemoglobin of 10.5 g/dL and a platelet count of 121/uL, hypertriglyceridemia of 356 mg/dL, and hyperferritinemia of 999 ng/mL. She underwent another bone marrow biopsy that showed hemophagocytosis. Due to concern for HLH, she had further workup significant for elevated serum soluble CD25 (IL2R alpha) at 4,380 pg/mL. She was diagnosed with HLH and a workup for an underlying etiology was initiated. Given concern for an underlying hematolymphoid malignancy, she had a positron emission tomography (PET) scan. This revealed mild mediastinal and bilateral hilar uptake in the lungs. The PET scan also found hepatosplenomegaly but did not show any abnormal uptake in the liver, or in any other organ system. Without a clear etiology for her HLH, the decision was made to repeat a transjugular liver biopsy. Results from the repeat liver biopsy revealed non-necrotizing granulomas in the parenchyma (Figure 2).

Given the patient’s prolonged constitutional symptoms, HSM, pulmonary findings in the PET scan, noncaseating granulomas, and absence of underlying malignancy, there was high suspicion for sarcoidosis. Ultimately, the decision was made to treat for sarcoidosis given its common predilection for the liver. She received pulse dose steroids consisting of 40 mg intravenous dexamethasone daily for four days. Her symptoms drastically improved and she was later discharged on a six month prolonged prednisone taper. Her profound and sustained response to steroid therapy further supported the underlying diagnosis of sarcoidosis. Two years later, she is still in complete remission doing well on low dose prednisone therapy of 5 mg daily.

## Discussion

Sarcoidosis is a systemic granulomatous disease primarily affecting the pulmonary and lymphatic systems [5]. While the diagnosis has been recognized for more than a century, many unanswered questions remain. The pathophysiology while largely unknown, is theorized to stem from an exaggerated granulomatous reaction to exposure of an unidentified antigen in susceptible patients [5]. The initial manifestations of sarcoidosis vary with demographic data, but most commonly include pulmonary symptoms, frequently as a persistent nonproductive cough [5]. Extrapulmonary sarcoidosis occurs in 30%-50% of patients and may occasionally be the initial disease presentation [5]. Hepatic manifestations, which occur in 20%-30% of patients, frequently present with constitutional symptoms [6] and may include hepatomegaly and rarely hepatic insufficiency [7]. Splenic manifestations are only seen in 10% of patients, frequently with splenomegaly and rarely with pancytopenia [7]. Sarcoidosis manifesting as MAS is a rare phenomenon seldom reported.

Historically MAS was primarily associated with systemic juvenile arthritis, with an estimated incidence of 7%-17% in the pediatric population [3]. In the largest study assessing occurrence of MAS with autoimmune diseases, Fukuya et al. looked at 1014 patients with autoimmune diseases seen at Hokkaido University Hospital from 1997-2007 and found 30 cases of MAS, primarily attributed to SLE, while no cases were attributed to sarcoidosis [8]. However, the absence of sarcoidosis attributed to MAS may be due to the low incidence of sarcoidosis in the Japanese population compared to European and African populations [5].

In an extensive literature review encompassing 421 patients in 117 papers of MAS in the rheumatologic population, Atteritano et al. found five (1.2%) cases that could possibly be attributed to sarcoidosis, two of which had active infections at the time of diagnosis [4]. A national survey conducted in France over two years found 26 patients with MAS, only one of which was related to sarcoidosis [9]. Other isolated case reports implicate sarcoidosis as a cause of MAS [10]. These studies and reports show that there is a rare albeit established association between MAS and sarcoidosis, and therefore it should be considered as a possible underlying etiology when working up a patient with suspected HLH/MAS.

Prompt diagnosis and treatment of the underlying condition precipitating MAS is necessary to prevent morbidity and mortality. Unfortunately the recognition of MAS is often difficult [11], and the nonspecific clinical findings are frequently attributed to other explanations. In our patient, the finding of a monoclonal CD8+ T-cell population required us to rule out an underling hematologic malignancy. The IHC and flow, however, did not show any evidence of malignancy. The TCR gene rearrangement findings were attributed to reactive non-malignant processes. A history of EBV exposure has been associated with lymphoproliferative disorders in pediatric and adult patients [12-13]. While she did report a history of infection in childhood, her serum EBV PCR remained negative throughout her hospitalization.

The Histiocyte Society published a set of guidelines to aid in the diagnosis of HLH (HLH-94) [14]. Five criteria were agreed upon for HLH including fever, bicytopenia, splenomegaly, hypertriglyceridemia, and hemophagocytosis. However, these criteria lack sensitivity and specificity. Hemophagocytosis, when present, may not be seen until later in the disease course [15], and even if present in high amounts, lacks diagnostic specificity [16]. These guidelines were updated in 2004 with the addition of three other criteria including soluble CD25 levels, serum ferritin level, and NK cell activity [14].

The management of HLH/MAS is primarily derived from studies in pediatric patients. Treatment for HLH is based on the HLH-94 protocol, which utilizes both chemotherapy and immunotherapy, starting with etoposide and dexamethasone, followed by maintenance with etoposide, dexamethasone, and cyclosporin A [17]. Intrathecal methotrexate was also used in patients with CNS involvement [17]. This may be followed by bone marrow transplantation in persistent, recurring or familial disease [17]. In the pediatric population, these treatment regimens resulted in 55% overall survival at three years and 62% survival with transplant [17]. Furthermore, in primary pediatric HLH, the addition of antifungal prophylaxis should be considered as fatal invasive fungal infections have complicated up to 50% of cases [18]. Regardless of the etiology, mortality is high in untreated HLH, with some literature indicating an average survival of a few months [14]. If an underlying cause is identified, clinicians should also focus on its appropriate treatment including antibiotics for infections and steroids for rheumatologic flare-ups. Interestingly, while biologic therapy such as tocilizumab and canakinumab have been monumental in the treatment of rheumatologic disease, their role in MAS is uncertain as several phase III studies have not shown any effect on MAS rates [19].

The mainstay of treatment for pulmonary sarcoidosis is systemic glucocorticoid therapy. Duration depends on the response, with most patients requiring a slow taper. If the disease is not controlled, antimetabolite therapy with methotrexate, azathioprine, leflunomide, or mycophenolate is used, followed by anti-tumor-necrosis factor agents like infliximab [20]. About 66% of patients undergo remission, with mortality at 5% usually from pulmonary complications [6]. The patient in this case required only steroids as the treatment of her underlying sarcoidosis with significant and sustained disease resolution.

## Conclusions

Macrophage activation syndrome (MAS) is a rare complication of sarcoidosis, and uncommonly, the initial presentation. This diagnosis can be challenging, especially since it is rarely encountered, and its findings frequently attributed to other phenomenon. However when disease manifestations are consistent with the HLH criteria, prompt workup is required to find the etiology and to tailor treatment. As such, sarcoidosis should be considered in the differential during a MAS/HLH workup.
